# Molecular and Clinical Advances in Understanding Early Embryo Development

**DOI:** 10.3390/cells12081171

**Published:** 2023-04-16

**Authors:** Lon J. Van Winkle

**Affiliations:** 1 Department of Medical Humanities, Rocky Vista University, Parker, CO 80122, USA; lvanwinkle@rvu.edu or lvanwi@midwestern.edu; 2 Department of Biochemistry, Midwestern University, Downers Grove, IL 60515, USA

## 1. Introduction

The articles in this Special Issue address a wide variety of topics concerning molecular and clinical advances in understanding early embryo development. For convenience, the papers presented in this editorial address topics from the examination of oocyte biology to the emergence of second cellular lineages in association with and following mammalian embryo implantation in the uterus. In addition, epigenetic contributions to reproduction, which may occur throughout pregnancy and in resultant offspring, are also considered.

## 2. Early Development

### 2.1. Oocytes

Four papers in this Special Issue consider molecular, clinical and even evolutionary advances in understanding oocyte development. A possible metabolic function of the Na^+^-dependent amino acid transport system B^0,+^ in porcine oocytes is the provision of amino acids for protein synthesis and leucine to initiate mTOR1 signaling [1]. This also appears to be the case in Xenopus oocytes, where B^0,+^ disappears prior to egg deposition in fresh water. This transporter appears not to be present in the oocytes of mammalian species exhibiting invasive embryo implantation in the uterus. Instead, B^0,+^ first appears in rat, mouse, and likely human embryos at the blastocyst stage (see description of function below). Contrastingly, B^0,+^ is highly expressed in sea urchin oocytes to take up amino acids from sea water, but only after they are fertilized [1]. These differences in the timing of B^0,+^ expression are an important mechanism of evolution known as heterochrony [1].

In mouse oocytes, there appear to be other needs for amino acid transport [2], which may also be the case for human oocytes [1]. For example, proline transport into mouse oocytes by the Na^+^-independent transporters, PAT1 and PAT2, decreases mitochondrial activity and the production of reactive oxygen species (ROS), and thus improves subsequent early embryo development after in vitro fertilization [2]. In humans, and likely other species, fertility may be altered by mutations in mitochondrial DNA in association with ovarian aging, possibly due to ROS production [3]. Interestingly, the replacement of damaged mitochondria by healthier counterparts in human oocytes may soon improve assisted reproductive technologies (ART) [4]. 

### 2.2. Preimplantation Development

Remarkably, our knowledge of the importance of amino acid transport, signaling, and metabolism to preimplantation development continues to develop. For example, in a paper of this Special Issue, Treleaven et al. report how proline transport into preimplantation mouse embryos fosters their development but in a stage-specific manner [5]. In other words, proline improves the preimplantation development of mouse zygotes, and the B^0^ amino acid transporter appears to be mainly responsible for this uptake on day 4 but not on other days of development [5]. Moreover, proline fosters development in a growth-factor-like manner, in part because proline can substitute for the apparent paracrine signaling among these embryos grown at a high density [1,6]. One or more proline transporter also seems to function as a transceptor to activate mTOR1, Akt, and ERK signaling in preimplantation embryos [1]. 

Similarly, glycine fosters the preimplantation development of mouse embryos in a hypertonic oviductal fluid-like medium, but it also antagonizes the positive effects of proline, possibly by inhibiting proline transport [1]. Of the amino acids, proline seems to be most beneficial for preimplantation development in vitro. Therefore, the most effective way to culture healthy mouse and possibly human embryos is to supply only proline in these media [5,6]. Although complex, conditions in vivo can also be highly suitable for fostering the development of the healthiest preimplantation embryos under physiologically normal conditions [1,7]. Hence, it still needs to be determined whether the latter conditions can be mimicked in vitro in ways that do not cause unwanted effects on offspring at birth or later in adult life [1]. 

As discussed by Chen et al. in this Special Issue [8], the detrimental effects of in vitro culture are significant for porcine embryos where only about 40% of fertilized porcine oocytes develop into blastocysts. While such cultures are considerably more successful in mouse and human embryos, children conceived through ART have much greater risks of developing metabolic syndrome and associated conditions, such as higher body fat, greater fasting blood glucose, hypertension, and cardiovascular disease, in comparison to children conceived under physiological conditions in vivo [7]. Supporting the notion that mimicking in vivo conditions can improve development in vitro, in this Special Issue, Saadeldin et al. report that extracellular vesicles from the endometrium can increase the frequency of attachment of porcine blastocysts [9]. Conversely, a model to mimic type 2 diabetes by culturing early rabbit embryos in a medium containing high levels of glucose and insulin showed extensive and unwanted initiation of a number of gene expressions in the resultant inner cell mass (ICM) and trophectoderm (TE) of blastocysts [10]. Hence, attempts to mimic physiological conditions in vitro can be complex, considering the possible variability of these conditions in prospective mothers. 

### 2.3. Implantation

Signaling and metabolic changes are also involved in initiating implantation and maintaining pregnancy in vivo. The uptake of leucine via amino acid transport system B^0,+^ initiates mTOR1 signaling in the TE of mouse and likely blastocysts of all mammalian species exhibiting invasive implantation [1]. This signaling leads to development of the trophoblast motility needed by blastocysts to invade the uterine epithelium. For unknown reasons, system B^0,+^ then becomes relatively inactive in blastocysts in utero probably due to the action of extracellular histones. However, it likely must be reactivated at the time of uterine penetration in order to help deprive T-cells of another B^0,+^ preferred substrate, tryptophan, thus preventing the rejection of blastocysts in implantation chambers [1]. At the same time, ICM cells are maintained in a pluripotent state, partly due to the trimethylation of lysine 4 in histone H3 to form H3K4me3 [1]. However, these ICM cells will soon begin to differentiate into a number of tissues including, eventually, those in the post-implantation embryo and fetus. 

### 2.4. Second Lineage Differentiation

Following differentiation of the ICM and TE from the morula, and in association with implantation, the TE begins to form extraembryonic tissues, such as the placenta, while the ICM gives rise to the primitive endoderm (hypoblast) and epiblast (EPI). Thus, second lineage differentiation begins, followed by endoderm, mesoderm, and ectoderm development from EPI cells. The yolk sac develops from hypoblast cells, whereas tissues of the embryo and fetus arise from the endoderm, mesoderm, and ectoderm. 

Several papers in this Special Issue concern these second lineage differentiations. Firstly, Duan et al. show that the silencing of a long terminal repeat element of an endogenous retrovirus, known as MacERV6-LTR1a, postpones the differentiation of TE, EPI, and hypoblast cells in cynomolgus monkey embryos at day seven [11], although the mechanism for this delay is still being explored. In the mouse, NANOG expression is needed for hypoblast formation, but Springer et al. demonstrate that this is not the case for bovine embryos [12]. Hence, species differences clearly exist in the mechanisms of these second lineage differentiations. The delineation of these differences is essential in species such as mouse and pig that are sometimes used as models for human embryo development. 

In addition to signaling molecules, epigenetic mechanisms likely play roles in regulating the emergence of these cell lineages in early mammalian embryo development, and the detailed mechanisms of these epigenetic modifications also vary among species. For example, the formation of the H3K4me3 needed to maintain ICM cell pluripotency in mice exclusively depends on threonine metabolism in mice, but in humans, H3K4 methylation likely relies on serine metabolism [1]. Further complicating this regulation are differences in chromatin structure in different cell lineages. As reported by Quan et al. in this Special Issue, domains have been identified as CpG-rich (forests) and CpG-poor (prairies) in chromatin [13]. In both early human and early mouse embryos, the ectoderm cell lineages show the weakest domain segregation, whereas the endoderm cell lineages display the strongest domain segregation in germ layers [13]. The significance of these chromatin domain segregations for epigenetic contributions to cell development is yet to be investigated. 

## 3. Epigenetics in Human Reproduction

The importance of epigenetics to human reproduction cannot be over emphasized. For example, in this Special Issue, Wen et al. report that the loss of *H19/IGF2* epigenetic imprinting in the decidual microenvironment of early human pregnancy is associated with recurrent spontaneous abortions [14]. More broadly regarding epigenetics and pregnancy outcome, diet-induced obesity in mice is associated with numerous methylation changes in DNA in genes associated with type 2 diabetes mellitus [15]. Additionally, offspring of such mice are also likely to be obese [16], probably due to the transmission of these epigenetic changes to offspring by their mothers. Importantly, a study in this Special Issue—to investigate whether a pre-pregnancy lifestyle intervention in obese women reduces the risk of obesity and cardiometabolic disease of their offspring—showed no effect of an intervention [16]. Moreover, these authors summarize numerous studies on humans and other animals that demonstrate little or no effect of such interventions on the health of offspring (Table 3 of [16]). Hence, the epigenetic changes associated with obesity and related conditions seem resistant to modification before transmission of the pertinent epigenetically modified genes to offspring via maternal germ cells.

## 4. Conclusions

- Amino acid transport and signaling in oocytes influence their mitochondrial metabolism, ROS production, and health; therefore, the replacement of damaged mitochondria in oocytes may soon improve ART in humans.
- During the preimplantation period, amino acid transport and signaling also foster more normal embryo development.
- It remains to be determined whether the healthiest preimplantation embryos develop in vitro in conditions that mimic the physiological environment in vivo or whether simpler conditions can also foster this development in vitro.
- Amino acid transport, metabolism, and signaling are also needed in blastocysts to maintain their pluripotent ICM cells and to foster the trophoblast invasion of the uterine epithelium during implantation in the uterus.
- The details of signaling needed to promote second cellular lineage differentiation in peri-implantation embryos varies among mammalian species used as models for human embryo development.
- Environmentally induced epigenetic changes in germ cells can render mammalian offspring less healthy, and these changes can be difficult to reverse.
